# Supplementary material for: Low-level Plasmodium vivax exposure, maternal antibodies, and anemia in early childhood: Population-based birth cohort study in Amazonian Brazil
Source: PLoS Negl Trop Dis. 2021 Jul 15;15(7):e0009568. doi: 10.1371/journal.pntd.0009568 (PMC8282015; doi:10.1371/journal.pntd.0009568)
Supplement: S1 Text — (PDF) [file pntd.0009568.s002.pdf]

# S1 Text: Supplementary Methods

Supplementary Material for:

## Low-level *Plasmodium vivax* exposure, maternal antibodies, and anemia in early childhood: population-based birth cohort study in Amazonian Brazil

**Multivariable analysis**

Negative binomial regression models were used to identify correlates of malaria risk among children. This analysis comprised all children assessed at delivery, except for those with unavailable API estimates for their areas of residence (n = 45). This gives a total of 1,494 children for whom malaria morbidity data from birth to their second birthday were retrieved from the case notification database, or 2,988 person-years of follow-up. Separate regression models were built for two continuous outcomes: (i) total number of laboratory-confirmed clinical malaria episodes among children, irrespective of the infecting parasite species, during their first two years of life and (ii) number of laboratory-confirmed vivax clinical malaria episodes among children during the same period, excluding mixed-species (*P. vivax* plus *P. falciparum*) infections. Models included sociodemographic, environmental, gestational, and neonatal covariates. Sociodemographic maternal covariates were: (i) wealth index stratified into quarters of approximately equal size, or quartiles (the first quartile comprises the interval between the smallest wealth index value and the median); (ii) years of schooling (<9, 10-12, or 12+ years); (iii) mother is beneficiary of the *Bolsa Família* conditional cash transfer program (no vs. yes); (iv) maternal skin color (white vs. non-white); (v) mother is economically active (has a paid job, no vs. yes); (vi) mother is the head of (economically responsible for) the household (no vs. yes);  and (vii) mother lives with a partner (no vs. yes). Environmental covariates were: (i) household size (1-2, 3-4, or 5+ people); (ii) housing materials (bricks vs. wood/mixed material); (iii) average API in the area of residence (continuous variable); (iv) source of drinking water in the residence (well, river, or mineral water), and (v) sewage disposal (rivers/streams vs. septic tank). Gestational covariates were: (i) gravidity (1, 2, 3, 4, or 5+), (ii) malaria during pregnancy

or at delivery (no vs. yes); (iii) use of micronutrient supplementation (multiple micronutrients, iron, folic acid) during pregnancy (no vs. yes); (iv) reported morbidity (hypertension, urinary infection, night blindness) and alcohol consumption during pregnancy (no vs. yes); (v) number of antenatal care visits attended (<6 vs 6+), and (vi) gestational weight gain (poor, adequate, or excessive). Neonatal covariates were: (i) birth weight (z-score, continuous variable); (ii) estimated gestational age at birth (<37, 37-41, or 42+ weeks), and (iii) type of delivery (vaginal vs. cesarean).

We run additional negative binomial regression models with the key adjustment covariate API stratified into quintiles: first quintile, 0.409 to 2.652 cases per 1,000 inhabitants; second, 2.738 to 10.398; third, 13.574 to 126.395; fourth, 130.101 to 414.67; and fifth, 435.74 to 1601.47. As shown in S1 Table, covariates associated with malaria in young children remain the same as above after adjusting for API stratified into quintiles instead of a continuous covariate.

We used survival analysis to test whether levels of specific maternal antibodies measured at delivery predicted vivax malaria risk in mothers and their children over the next 12 or 24 months. We used Cox proportional hazards models to compare hazard ratios (HRs) for the time to the first vivax malaria episode after delivery in mothers and the first malaria episode experienced by their children (n = 1,095) across quintiles of maternal antibody levels, while adjusting for potential confounders listed in the footnote of Table 2. The first quintile (reference) comprises 20% of the study mothers with the lowest RIs. We used the hierarchical framework of disease determination described in S2 Fig. to select for covariates to be retained in the final multiple models.

We next tested whether previous malaria episodes in children were associated with an increased risk of anemia at the age of 2 years (dichotomic outcome variable). This analysis comprised 860 children who had hemoglobin measurements at the age of approximately 2 years (average, 725.87 days; standard deviation, 44.46 days). Covariates included in the multiple logistic regression models were essentially those listed under the malaria risk models

and some additional ones: (i) child's age (in days) at the time of the 2-year follow-up assessment (to account for individual age variation, since children were not exactly 2 years old); (ii) child's sex (male vs. female), (iii) area of residence (rural vs. urban); (iv) mother's age; (v) maternal social support network; (vi) maternal anemia (hemoglobin concentration <110 g/L) at delivery (no vs. yes); (vii) child currently attends daycare (no vs. yes); (viii) child regularly exposed to the sun (no vs. yes); (ix) duration of total breastfeeding (months, continuous variable), (x) current use of iron, vitamin, or folic acid supplements (no vs. yes); (xi) child morbidity since birth (pneumonia, dengue, gastrointestinal bleeding, or helminth infections; no vs. yes), (xii) recent child morbidity (diarrhea, vomiting, or flu-like illness within the past 15 days; no vs. yes), (xiii) hospitalization since birth (no vs. yes), and (xiv) malaria episode (any species) since birth. The hierarchical model for variable selection is shown in S3 Fig. To explore the impact of the frequency and timing of malaria episodes, as well as the infecting malaria parasite species, we considered four malaria exposure categories among children in separate models: (i) number of laboratory-confirmed malaria episodes during the first two years of life, caused by any species (0, 1, or 2+), (ii) number of laboratory-confirmed vivax malaria episodes during the first two years of life (0, 1, or 2+), (iii) one or more malaria episodes, any species, ≤12 months before the follow-up visit (no vs. yes), and (iv) one or more vivax malaria episodes ≤12 months before the follow-up visit (no vs. yes). Estimates of odds ratios along with their 95% CIs were interpreted to reflect the magnitude of the observed association between malaria exposure and anemia while controlling for all other covariates.
