# Supplementary material for: Low-level Plasmodium vivax exposure, maternal antibodies, and anemia in early childhood: Population-based birth cohort study in Amazonian Brazil
Source: PLoS Negl Trop Dis. 2021 Jul 15;15(7):e0009568. doi: 10.1371/journal.pntd.0009568 (PMC8282015; doi:10.1371/journal.pntd.0009568)
Supplement: S1 Table — In these models, API was entered as a categorical variable (stratified into quintiles). (PDF) [file pntd.0009568.s003.pdf]

S1 Table. Multiple negative binomial regression analysis of correlates of malaria (all species) and vivax malaria from birth to two years of age in children from the MINA-Brazil birth cohort study (n = 1494). In these models, API was entered as a categorical variable (stratified into quintiles).

		Negative binomial regression models							
		Outcome: all malarias (n = 251)				Outcome: vivax malaria (n =224)			
**Covariate**	**n[a]**	**IRR[b]**	**(95% CI)[c]**		**P**	**IRR[b]**	**(95% CI)[c]**		**P**
**Wealth index quartile**									
1st (poorest)	341	Reference				Reference			
2nd	358	0.640	(0.342	1.200)	0.164	0.665	(0.349	1.268)	0.215
3rd	363	0.734	(0.386	1.393)	0.344	0.790	(0.408	1.530)	0.485
4th	364	0.174	(0.063	0.485)	0.001	0.206	(0.074	0.577)	0.003
**Beneficiary of Bolsa Família[d]**									
no	821	Reference				Reference			
yes	605	1.916	(1.110	3.309)	0.020	1.879	(1.073	3.290)	0.027
**API[e]**									
1st (lowest)	300	Reference				Reference			
2nd	312	2.653	(0.840	8.379)	0.096	3.132	(0.908	10.803)	0.071
3rd	292	2.646	(0.840	8.340)	0.097	3.319	(0.967	11.396)	0.057
4th	323	4.701	(1.571	14.064)	0.006	4.982	(1.503	16.513)	0.009
5th	267	20.719	(7.093	60.524)	<0.001	22.731	(7.078	73.007)	<0.001
**Malaria in pregnancy**									
no	1316	Reference				Reference			
yes	178	2.203	(1.207	4.019)	0.010	2.386	(1.295	4.396)	0.005
**Mother´s gravidity**									
primigravidae	570	Reference				Reference			
2	365	0.281	(0.130	0.606)	0.001	0.274	(0.123	0.610)	0.002
3	203	0.721	(0.341	1.527)	0.393	0.783	(0.366	1.674)	0.528
4	118	0.568	(0.226	1.433)	0.231	0.498	(0.191	1.296)	0.153
>= 5	170	1.042	(0.499	2.172)	0.913	1.094	(0.516	2.317)	0.815

[a]Totals across exposure categories may not equal 1494 because of missing

[b]IRR = Incidence rate ratio.

[c]CI = confidence interval.

[d]Bolsa Família = Federal conditional cash transfer program.

[e]API = annual parasite incidence in the locality of residence.
