# Supplementary material for: Low-level Plasmodium vivax exposure, maternal antibodies, and anemia in early childhood: Population-based birth cohort study in Amazonian Brazil
Source: PLoS Negl Trop Dis. 2021 Jul 15;15(7):e0009568. doi: 10.1371/journal.pntd.0009568 (PMC8282015; doi:10.1371/journal.pntd.0009568)
Supplement: S2 Table — (PDF) [file pntd.0009568.s004.pdf]

S2 Table. Adjusted β coefficients and bootstrap 95% confidence intervals (CI) of malaria exposures associated with hemoglobin concentration (g/L) at two years of age, as estimated using quantile regression analysis for children from the MINA-Brazil birth cohort study.

	**Quantile of hemoglobin concentration at 2 years (n = 860)**		
**Malaria exposure**	**Percentile 25th**	**Percentile 50th**	**Percentile 75th**
**Malaria since birth, any species:**			
Overall	**-2.99 (-7.17; -1.19)**	-2.23 (-7.65; 3.20)	-2.95 (-6.52; 0.62)
1 malaria	4.41 (-2.99; 11.82)	1.62 (-3.03; 6.27)	**-2.95 (-5.34; -0.57)**
2+ malarias	**-5.42 (-10.42; -0.41)**	-6.27 (-14.28; 1.74)	-5.60 (-16.50; 5.18)
**Vivax malaria since birth:**			
Overall	-2.99 (-7.41; 1.43)	-2.25 (-7,17; 2.67)	-2.77 (-6.50; 0.96)
1 vivax malaria	5.05 (-2.57; 12.67)	1.12 (-2.92; 5.17)	-2.77 (-6.64; 1.10)
2+ vivax malarias	**-5.10 (-9.71; -0.50)**	**-6.35 (-12.44; -0.26)**	-5.65 (-18.61; 7.32)
**Malaria within the last 12 months, any species**	-3.10 (-8.94; 2.74)	-3.04 (-10.11; 4.03)	-2.82 (-6.91; 1.26)
**Vivax malaria within the last 12 months**	-3.00 (-7.21; 1.21)	-3.82 (-9.09; 1.45)	-3.15 (-8.23; 1.94)

Qualtile regression models adjusted for: child age and sex, wealth index, whether the mother is beneficiary of the Bolsa Família program (no vs. yes), maternal schooling, whether the mother is the household head, household size, maternal anemia at delivery, and gestational age. Bold font indicates $P < 0.05$.
