# Supplementary material for: Low-level Plasmodium vivax exposure, maternal antibodies, and anemia in early childhood: Population-based birth cohort study in Amazonian Brazil
Source: PLoS Negl Trop Dis. 2021 Jul 15;15(7):e0009568. doi: 10.1371/journal.pntd.0009568 (PMC8282015; doi:10.1371/journal.pntd.0009568)
Supplement: S1 Fig — The continuous diagonal line represents identical antibody levels in maternal and cord-blood samples. Spearman correlation coefficients were 0.814 for anti-PvAMA1 antibodies, 0.801 for anti-PvDBP antibodies, and 0.858 anti-PvMSP119 antibodies (P<0.0001 for all). (PDF) [file pntd.0009568.s005.pdf]

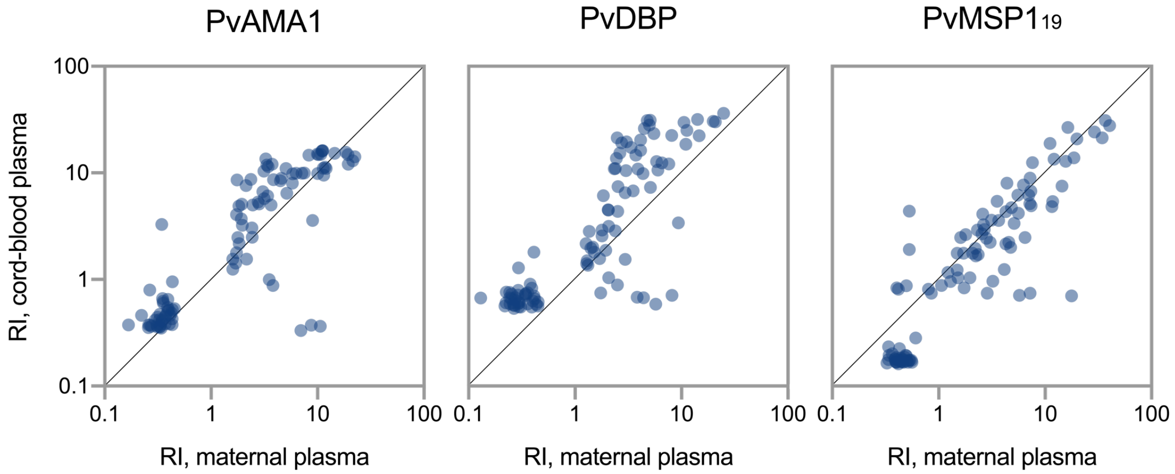

S1 Fig. Levels of specific antibodies (reactivity indices in log scale) in 101 paired maternal and cord-blood plasma samples from MINA-Brazil study participants. The continuous diagonal line represents identical antibody levels in maternal and cord-blood samples. Spearman correlation coefficients were 0.813 anti-PvAMA1 antibodies, 0.801 for anti-PvDBP antibodies, and 0.858 anti-PvMSP1$_{19}$ antibodies (*P*<0.0001 for all).
