# Supplementary material for: Low-level Plasmodium vivax exposure, maternal antibodies, and anemia in early childhood: Population-based birth cohort study in Amazonian Brazil
Source: PLoS Negl Trop Dis. 2021 Jul 15;15(7):e0009568. doi: 10.1371/journal.pntd.0009568 (PMC8282015; doi:10.1371/journal.pntd.0009568)
Supplement: S2 Fig — (PDF) [file pntd.0009568.s006.pdf]

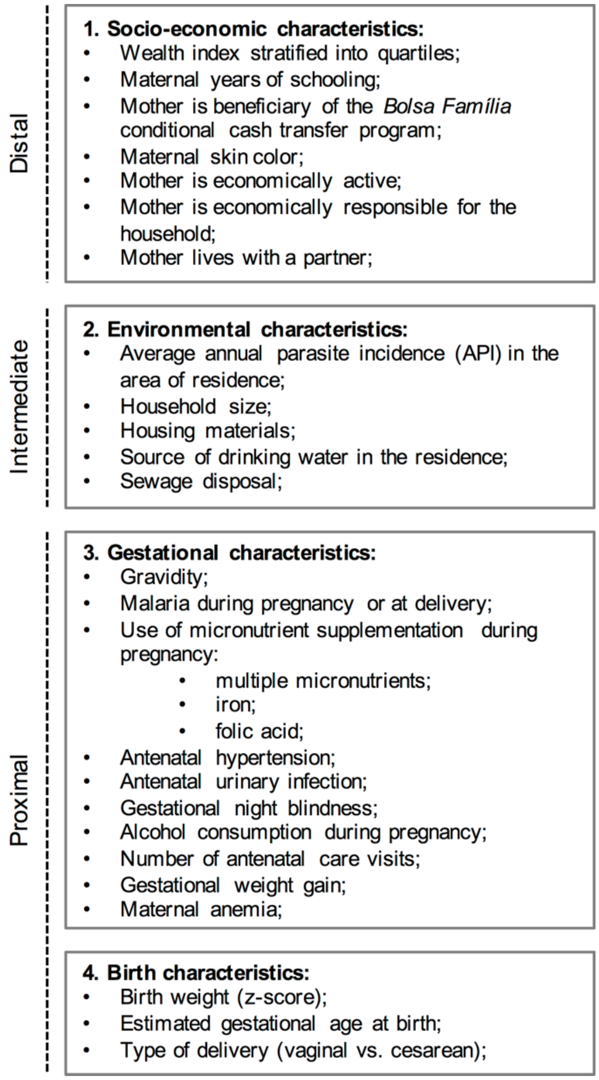

S2 Fig. Hierarchical conceptual framework for selection of correlates of malaria risk in early childhood.
