# Supplementary material for: Low-level Plasmodium vivax exposure, maternal antibodies, and anemia in early childhood: Population-based birth cohort study in Amazonian Brazil
Source: PLoS Negl Trop Dis. 2021 Jul 15;15(7):e0009568. doi: 10.1371/journal.pntd.0009568 (PMC8282015; doi:10.1371/journal.pntd.0009568)
Supplement: S3 Fig — (PDF) [file pntd.0009568.s007.pdf]

Child's sex and age

Distal

1. Socio-economic characteristics:
- Wealth index stratified into quartiles;
- Maternal years of schooling;
- Mother is beneficiary of the *Bolsa Família* conditional cash transfer program;
- Maternal skin color;
- Mother is economically active;
- Mother is economically responsible for the household;
- Mother lives with a partner;
- Area of residence (rural vs. urban);

Intermediate

2. Environmental characteristics:
- Household size;
- Source of drinking water in the residence;
- Sewage disposal;

3. Maternal characteristics:
- Mother's age;
- Gravidity;

4. Gestational characteristics:
- Malaria during pregnancy or at delivery;
- Use of micronutrient supplementation during pregnancy:
    - multiple micronutrients;
    - iron;
    - folic acid;
- Antenatal hypertension;
- Antenatal urinary infection;
- Gestational night blindness;
- Alcohol consumption during pregnancy;
- Number of antenatal care visits;
- Gestational weight gain;
- Maternal anemia;

5. Birth characteristics:
- Birth weight (z-score);
- Estimated gestational age at birth;
- Type of delivery (vaginal vs. cesarean);

6. Behavior characteristics:
- Social support network;
- Child currently attends daycare;
- Child regularly exposed to the sun;

Proximal

7. Nutritional characteristics:
- Duration of total breastfeeding;
- Current use of iron, vitamin, or folic acid supplements;

8. Child health:
- Pneumonia since birth;
- Dengue since birth;
- Gastrointestinal bleeding;
- Helminth infections;
- Diarrhea within the past 15 days;
- Vomiting within the past 15 days;
- Flu-like illness within the past 15 days;
- Hospitalization since birth;
- Malaria episode (any species) since birth;

S3 Fig. Hierarchical conceptual framework for selection of correlates of anemia risk in early childhood.
