# Supplementary material for: Low-level Plasmodium vivax exposure, maternal antibodies, and anemia in early childhood: Population-based birth cohort study in Amazonian Brazil
Source: PLoS Negl Trop Dis. 2021 Jul 15;15(7):e0009568. doi: 10.1371/journal.pntd.0009568 (PMC8282015; doi:10.1371/journal.pntd.0009568)
Supplement: S4 Fig — Bars show the number of laboratory-diagnosed malaria episodes per child over the first two years of life (empirical data) and the continuous line shows the negative binomial function fitted to data. Both panels show the same data, but y-axis values in the right panel are square root-transformed to improve data visualization. (PDF) [file pntd.0009568.s008.pdf]

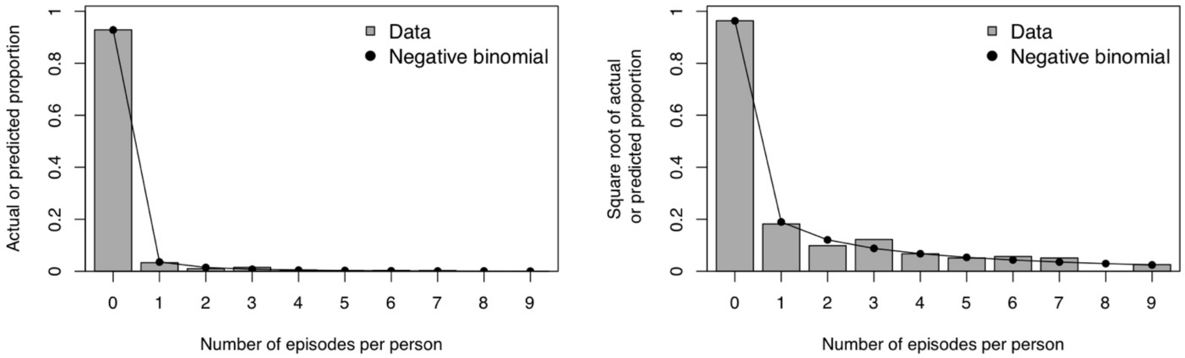

S4 Fig. Frequency distribution of the number of malaria episodes in the study population. Bars show the number of laboratory-diagnosed malaria episodes per child over the first two years of life (empirical data) and the continuous line shows the negative binomial function fitted to data. Both panels show the same data, but y-axis values in the right panel are square root-transformed to improve data visualization.
