# Supplementary material for: Low-level Plasmodium vivax exposure, maternal antibodies, and anemia in early childhood: Population-based birth cohort study in Amazonian Brazil
Source: PLoS Negl Trop Dis. 2021 Jul 15;15(7):e0009568. doi: 10.1371/journal.pntd.0009568 (PMC8282015; doi:10.1371/journal.pntd.0009568)
Supplement: Attachment — Submitted filename: PLOS NTD review.docx [file pntd.0009568.s009.docx]

**Overall comment**

The paper summarises findings of birth cohort in a malaria-endemic setting in the Brazilian Amazon. The paper contains a lot of interesting data on P. vivax infection that is very much worth publication. However, the current format is relatively difficult to digest. This is because the paper focusses on a number of outcomes (not all of them are immediately clear), and uses a wide range of analytical approaches. It effectively reads like a combination of three small somewhat related papers merged into one. It reports on the burden of postpartum clinical malaria in the mother, the burden of clinical malaria in the first two years of life of the child, the association between gestational malarial infection (?PCR) and clinical malaria in the child, the relationship between P. vivax infections in the first two years of life and anaemia at Year 2, amongst others.

I recommend to focus the story of the paper on *P. vivax* infections during the first 2 years of life (90% of infections were vivax infections), and factors associated with these infections, in particular maternal *P. vivax* status. Less is more when it comes to telling a consistent and interesting story, which this is. Telling the story would be strengthened by more clearly highlighting how malaria during pregnancy was diagnosed (and species breakdown), and restricting analyses to mother -infant pairs for whom you were able to determine the API, maternal infection status, and clinical malaria follow-up for 2 years in children. Relationships between levels of three anti Pv antibodies measured in maternal blood and P.v. in first two years of life could be integrated into the story, but the story of anaemia is probably more relevant. In addition to API, adjusting analyses of the association between gestational Pv with infant Pv for maternal postpartum clinical vivax episode may help to deliver some further insights regarding the mechanisms of the aforementioned association.

There is a parallel story of maternal postpartum malaria, and the relationship between levels of three anti Pv antibodies measured in maternal blood at birth and maternal postpartum malaria and infant malaria. This complicates the paper and would probably be best told as a stand-alone paper or in a supplemental file, highlighting how pre and postpostum infection risk compares in mothers. Figure 3 A is helpful though in a way. The main issue with these data to is the lack of regular PCR-based screening for infection during and after pregnancy, which precludes a conclusive analysis of P. vivax risk in pregnant versus postpartum women. The measured antibodies are more likely to represent a marker of current or recent infection in others (and thus a predictor of relapse in mothers).

Include a STROBE guideline tick chart as a supplementary file and ensure the paper is formatted accordingly.

Include a Table (new Table 1) summarising the main characteristics of your cohort.

Ensure that your malaria measures are clearly explained (differentiate between infection and clinical malaria).

Rationalise the outcome measures for your study and how they were assessed – there are a great range of them, ORs, IRR, cumulative incidence, HRs etc etc. – the are all reasonable but need to be presented in a more coherent manner.

The paper needs some grammar editing.

**Abstract**

Overall comment:

The methodology section needs to be strengthened, e.g. how and when was malaria assessed, details of antibody assays and time points, surveillance frequency etc. The background section could include a mention of the lack of knowledge around the relationship between P. vivax malaria in pregnancy and risk of P. vivax infection in infants.

- Malaria causes significant morbidity and mortality in under-five children in sub-Saharan

Africa and the Asia-Pacific region, but neonates and young infants remain relatively

protected from clinical disease putatively due to the transplacental transfer of maternal

antibodies.

Comment: Suggest to make two sentences out of this to improve readability

- Plasmodium vivax malaria in early childhood – traditionally viewed as

a benign infection…

Comment: Are you referring to the more global statement of vivax being a ‘benign’ malaria, or specifically to P. vivax infections in early childhood being viewed as benign – pls clarify.

- We show that only 7.1% of the >1,500 participants in a birth cohort study..

Comment: clarify participants = children; 7.1% (n=?); >1,500 – put precise number here.

- Line 50-51 How was malaria in pregnancy defined
- Line 48 children rather than toddlers
- Line 49 – few children (1.4%)
- Line 50-51 – put some stats here to support your argument. State that you adjusted for the transmission setting shared by mother and child – this is good!
- Line 52 present your antibody findings in a separate statements – levels of these three antibodies did not associate with risk of P. vivax at 2 years. Not enough infections in first year of life to clearly state if they had an impact there.
- Line 57 – The conclusion read like a conclusion for this paper in combination of the previously paper published paper by the same group. It should instead focus on findings from this study only.
- Line 59 – Maternal infection, the majority being P. vivax, associated with an increased risk of infants getting treated for clinical P. vivax malaria to local health centres. Then add your thoughts on what this means for prevention of malaria in pregnancy and infancy in your setting.

**Introduction**

Overall comment:

I would remove mention of the ‘results’ in the last paragraph of this background section. I would include more information on research that has assessed relationships between malaria in pregnancy and malaria in infants, including their methodological shortcomings to adjust for shared exposure risk. Highlight the lack of P. vivax studies on this subject. If available, I would also mention any studies that looked at maternal P. vivax antibodies and any protective effects for the offspring, should you decide to keep this.

- Line 95-96 and appears to be more resilient than P. falciparum to current control

Comment: Do the authors want to comment as to why they think this is? I think it matters!

- Line 108

Comment: I would state that P. vivax is the most widespread malaria parasite globally: <https://www.ncbi.nlm.nih.gov/pmc/articles/PMC5198891/>

- Line 117: which continent?
- Line 117-119

Comment: Clarify that the scope of the study. There are many aims – characterize the burden of malaria in the first two years of (ex-utero) life; assess relationship between MIP and risk of infant malaria; assess relationship between maternal anti-P. vivax antibodies and risk of infection in infants; association of P. vivax and anaemia at age 2

**Materials and Methods**

Overall comments:

It would be good to state more how each statistical approach addresses a particular aim of the study. It was challenging to work out what the different analyses were addressing. The core of this is to clearly address and refine the aims first (see above).

Please provide some background details on malaria prevention and detection strategies during pregnancy, as well as after pregnancy, in your study settings. What is the relapse timing and frequency of *P. vivax* in your setting? How does it compare to other settings?

The description of covariates is lengthy and complicated, especially as you are using different covariates for your analyses. This could be rationalised

Line 137 – Mother-baby pairs were enrolled during pregnancy at public antenatal clinics, or at birth etc…

146 – API, number of laboratory-confirmed clinical malaria cases.

Line 150 – this link should go into the reference section. How does this infant mortality compare to the rest of the country.

Line 158-160 – Please clarify: a) PCR assay used; PCR was done for all mothers; c) PCR was done on which blood – maternal peripheral, placental etc - you cannot expect the reader to go through your previous publications.

Line 161 – clarify make of baby scale – you subsequently never mention birthweight again so wondered whether a) birthweight should be included in your analyses as it may affect malaria risk – if you decide not too you can as well remove this bit.

Line 162-164 – reword this so it’s a bit clearer what happened to the rural women/infants.

176 – 178 – can you confirm if these data refer to cases in symptomatic infants/women – that’s how I understand it so perhaps add ‘computed microscopy-confirmed clinical malaria episodes… etc

211 – 213 – Can you clarify here that you did not lump these together for your analyses but looked at correlation between maternal and infant levels (this only becomes clear in the results section)

234 – 236 is it the cumulative incidence (birth to age 2) that you were calculating?

238 – please clarify who these participants are – kids or mums. Do this throughout the manuscript.

241 – if you make infant malaria risk a main outcome of your study, then it might be easiest and clearest to restrict all analyses to this group (i.e. mother infant pairs for whom you can establish an API).

241 – 244 you state here that regression models were built for continuous outcomes total number of lab confirmed malaria episodes, and lab confirmed vivax episodes. I have struggled to subsequently find these analyses in the manuscript. Do you mean here that you calculated IRR of any clinical malaria, or P. vivax malaria, as the ratio of clinical malaria episodes in children per person-year by maternal infection. Could be a lot clearer

245 –Would maternal smoking status be relevant? Sex of the infant has been reported to modify malaria risk so that may also need to be considered. Bed net use is also key but not reported – discuss this in your limitations section. I mentioned birthweight earlier.

252 – is API normally distributed?

269 – is this IRR calculated from a ratio of the number of episodes per person-year by maternal gestational malaria status? Clarify the denominator

273 – I would focus on infants. Introducing the story the association between anti-Pv antibodies in the mother at birth and protection from subsequent infection in the other is interesting but diverts away the attention from the core part – impact on infection risk in infants. Postpartum infections in the mother could be used as a covariate in your multivariable analyses, as a second marker of shared exposure risk (and for vivax, relapses in the mother may be the primary course of infection in their kids).

281 – previous malaria episodes – in the child?

283 – when you say ‘participants’ spell out which participants you mean – children or mothers

285 – why include the child’s age in days as a covariate at the 2 year follow – the child should be 2 years old.

295 – 303 – clarify that this is malaria in the infant/child, and that these categories were used to determine relationships between malaria infection in children.

**Results**

311-312 – are you trying to say that all these episodes were ‘uncomplicated disease episodes (defined how)’?

312 – 314 – I would remove the maternal data here and focus on infant malaria story first.

316 – first sentence – remove – this is stuff for the discussion.

342 – Malaria in pregnancy – can you spell out in your results section how many women were infected at birth (it’s in the table but needs to be moved to the text), and the main species of maternal infection at birth.

343 – 348 this would probably fit in the discussion section. Instead, it would be interesting to see the data on maternal postpartum infection and its relationship with infections in children.

358 – 362 – this could almost be put in the methods. It attempts to validate the notion that maternal levels reflect cord blood (infant) antibody levels (levels are usually higher in cord blood compared to mat plasma), which then helps you to comment on the relationship between maternal antibody levels and risk of infection in infants.

367 – we noted

367-368 we noted an increased risk of postpartum clinical vivax malaria in mothers with high antibody levels (quintile 5).

371- 373 – for discussion – just present the data here. You will need to discuss radical cure, relapse frequency characteristics etc for our settings.

366 – 376 – please mention the fact that anti Pv Ab levels are not associated with infant malaria (DBP maybe?). All you mention here is that you have not done this analysis for infants, and we have to dig in the table to find out what happened to the children.

384-385 – define anaemia in the methods section. What are your exposures, what are you outcomes – this needs to be clearly stated in the methods.

387-388 define Bolsa family as a poverty indicated in methods, and then use poverty throughout in your results. Ref to your Supp Fig 2 here for the reader to be reminder what covariates you looked at. Would maternal gestational malaria be relevant?

**Discussion**

Overall comment:

I would summarise the main findings of the overall study in the first paragraph, and then move on to discuss the individual points/discoveries.

Line 417 – here we show that clinical vivax malaria etc…

418 and following – how do you explain your findings – you are only suggesting that the low incidence of *P. vivax* malaria in the first year of life may not be entirely mediated by maternal antibodies or fetal haemoglobin, the protective ability of which you suggest weans after the first six months. The references used in this paragraph largely related to falciparum infections.

424-431 Explain the findings of your study in the context of the published data of P. vivax antibody studies and protection from infection. Focusing on research that investigates the role of passive immunity for Pv would be most interesting. Are your antibody targets markers of maternal infection (including latent infections) rather than those components of the immune system that are associated with protection from infection? They associated with recurrence in the mother, probably due to relapse.

433 – 440 this would fit with the paragraph staring at line 418

442 – 453 This to me is one of the key findings of the study, and the big novelty, as you are showing it for *P. vivax*. I would expand this analysis to see if this association between malaria during pregnancy and malaria in the child remains if maternal postpartum malaria was included in the model.

460 – we note that hb measurements were taken in >70% of the children in our cohort at the 1 year etc… and X % of children were anaemic and thus supplemented.

465 – merge this paragraph with the subsequent one, and abbreviate things somewhat.

480 – highlighting the biology of *P vivax* in your introduction would be good, as it shapes our thinking around the origin of blood stage infections in the mother, and the likely source of infection for the child.

482 cause rather than originate. You need to present data on relapse frequency for your setting if you can.

484 – 487 This issue here is that the mother should be treated, and there are restrictions around this. Your data suggests that children are ok up until one year.

Limitations: mention sample size; missing data; multiple comparisons; single time point of screening for infection in all women (delivery; lack of documentation of radical cure in mothers or infants. This issue with not having much P. falciparum is not a major limitation – your strength is that you are looking at Pv, for which there is not much such data.

**Conclusion**

The first two sentences should probably go into introduction rather than be named here. This section focusses on the findings of this analysis.

508-512 – split sentence into 2

512 – you are not showing the data on anaemia and impaired infant growth so I would state ‘which has been associated with deleterious impacts on child growth and development’.

Offer a way forward how you suggest the problem can be prevented, based on your findings. What can be done to protect young children to get *P. vivax*? Etc.

**Table 1**

**Footnote a** – discuss this in more detail in the methods section. There you mention creating a variable for missing data, so this is confusing.

This table/analysis could including maternal postpartum infection as a variable.

Figure 3 – here you used episodes per person-month – what do you use to calculate the IRRs. Define incidence in your figure cap.
