# Supplementary material for: Low-level Plasmodium vivax exposure, maternal antibodies, and anemia in early childhood: Population-based birth cohort study in Amazonian Brazil
Source: PLoS Negl Trop Dis. 2021 Jul 15;15(7):e0009568. doi: 10.1371/journal.pntd.0009568 (PMC8282015; doi:10.1371/journal.pntd.0009568)
Supplement: Attachment — Submitted filename: Reviewers.docx [file pntd.0009568.s010.docx]

We thank all reviewers for carefully reading the manuscript and providing a number of suggestions to improve it, which are highlighted in blue. We provide detailed responses to each reviewer comment or suggestion.

**Reviewer's Responses to Questions**

**Methods**

Reviewer #2: I have listed by comments pertaining to the methods in the Editorial and Data Presentation Modifications and Summary and General Comments sections of this review. Overall the methods are fairly well described, but more attention should be paid to their limitations as I have outlined.

**Results**

Reviewer #2: I have listed by comments pertaining to the results in the Editorial and Data Presentation Modifications and Summary and General Comments sections of this review.

--------------------

**Conclusions**

Reviewer #2: I have listed by comments pertaining to the conclusions in the Editorial and Data Presentation Modifications and Summary and General Comments sections of this review. I have highlighted areas where I believe the limiations of the study need to be emphasised more.

--------------------

**Editorial and Data Presentation Modifications?**

Reviewer #2: L45: Specify the actual number rather than just >1,500.

Done.

L94: Please check the 800,000 number in the WHO report, a quick look suggests it should be 889,000 (822,000-970,000) but I may be wrong.

You are absolutely correct: the 800,000 estimate came from the 2019 report (data for 2018). The latest estimate (2020 report) is 889,000. Corrected (L 115).

L100-103: Worth mentioning here that reduced exposure to vectors is also a proposed mechanism.

Done.

L119-125: These are results, suggest highlighting the primary research question(s) of the study here instead.

Done. The last two sentences of the Introduction now read:

“Here we examine the burden of early life malaria in Juruá Valley, an Amazonian hotspot in northwestern Brazil that contributes nearly 18% of the country′s malaria cases [15]. We describe the incidence of *P. vivax* infection during the first two years of life in a prospective birth cohort study and its association with maternal antibodies and anemia at the age of two years” (L 146-150).

L165-166: The reference does not support this assertion and highlights that the poorest families were most likely to be lost to follow-up: “Compared with participants at birth, the proportion (95% CI) of participants from poorest families declined from 24.9% (22.5 to 27.4) at birth to 19.3% (16.8 to 22.1) at 2 years”. This should be acknowledged.

You are absolutely correct. We changed the text accordingly:

“Here we analyze information collected during the follow-up visit at the age of 2 years. Participants in the assessment and those who were lost to follow-up had similar perinatal health profiles, but the proportion of participants from poorest families declined from 24.9% at birth to 19.3% at 2 years, mostly due to the exclusion of children from the remote rural sites [16].” (L 214-218)

L245-246: Quartiles are the 25th, 50th and 75th percentiles. If you cut the data at the quartiles you stratify it into fourths.

Thank you for the comment. We agree that the meaning of quartile is somewhat ambiguous, as it denotes both (a) the 25th, 50th and 75th percentiles that divide data into four classes of equal size and (b) each of these classes. We use the second definition of quartile from the Merriam-Webster dictionary (https://www.merriam-webster.com/dictionary/quartile):

“Definition of quartile

: any of the three values that divide the items of a frequency distribution into four classes with each containing one fourth of the total population

also : any one of the four classes”

We changed the text to make it clear that we use quartile with the second meaning:

“Sociodemographic maternal covariates were: (i) wealth index stratified into quarters of approximately equal size, or quartiles (the first quartile comprises the interval between the smallest wealth index value and the median)…” (S1 Text).

L245-263: What was the assumed functional form of the relationship between continuous covariates and the outcome? Linear?

Our final negative binomial regression models have only one continuous variable: API. The expected value of the dependent variable is given in the models as a linear function of the predictor covariates, including API. This may not be the case of API, a key covariate for adjustment. To explore this, we did an additional analysis using APIs stratified into quintiles (first quintile, 0.409 to 2.652; second, 2.738 to 10.398; third, 13.574 to 126.395; fourth, 130.101 to 414.67; fifth, 435.74 to 1601.47). The models are described in S1 Text and results are shown in S1 Table. In conclusion, the main association observed, between maternal malaria in pregnancy and subsequent risk of malaria in children, remains unaltered when adjusting for the intensity of malaria transmission in the locality using API stratified into quintiles.

L267-268: Can the authors further explain how a missing value category is incorporated into a continuous covariate?

The complete list of variables introduced into our negative binomial regression models is now provided in S1 Text in addition to S2 Fig., along with information regarding the type of variable (whether categorical or continuous). We have only two continuous variables: API and birth weight (z-scores). Individuals with missing API (a critical covariate for adjustment) were excluded from the adjusted models. Birth weight was not selected for inclusion in the final model (Table 1), hence no individual was excluded from the adjusted analysis on the basis of missing birth weight measurements.

To make the text clearer, we introduced the following change (bold):

“Participants with missing values **in categorical covariates** were maintained in the model by creating a new missing-value category.” (S1 Text)

L276 – quintiles are the 20th, 40th, 60th and 80th percentiles. I think the authors are referring to “fifths”. Can the authors also justify their choice of categorising the antibody exposure into fifths rather than retaining the continuous information?

Similar as above. We acknowledge that “quintile” has two different meanings and this may cause ambiguity (https://www.merriam-webster.com/dictionary/quintile):

“Definition of quintile

: any of the four values that divide the items of a frequency distribution into five classes with each containing one fifth of the total population

also : any one of the five classes”

To make it clear that we refer to the second meaning, we changed the text to:

“We used Cox proportional hazards models to compare hazard ratios (HRs) for the time to the first vivax malaria episode after delivery in mothers and the first malaria episode experienced by their children (n = 1,095) across quintiles of maternal antibody levels, while adjusting for potential confounders. The first quintile (reference) comprises 20% of the study mothers with the lowest RIs.” (S1 Text)

We also changed Table 2 title (bold) to:

“Cox proportional hazards models for time to the first vivax malaria **according to quintiles of antimalarial antibody levels** in mothers between 0 and 12 months after delivery [n = 1095] and between 12 and 24 months after delivery [n = 981]) and children (24 months of follow-up since birth; n = 1096) from the MINA-Brazil birth cohort study.”

We also indicated, in Table 2, that the reference group is the first quintile (i.e., mothers with the lowest antibody levels).

L286 – typo: “area or residence”

Thanks, corrected!

L328 – Can the authors clarify if an infection of both P. vivax and P. falciparum considered as a P. vivax infection in the data analysis?

Only *P. vivax*-only infections were considered as “vivax malaria” in our analyses. To make it clear, we introduced the following (bold):

“Separate regression models were built for two continuous outcomes: (i) total number of laboratory-confirmed malaria episodes, regardless of the infecting parasite species, during the first two years of life and (ii) number of laboratory-confirmed vivax malaria episodes during the same period**, excluding mixed-species (*P. vivax* plus *P. falciparum*) infections**.” (L 330-340).

Figure 5A: Why not display antibody RI on the y axis and continuous age on the x axis?

Please see below. We did generate scatterplots as suggested, showing RI on a linear or log scale. However, we argue that the age-prevalence histograms shown in Figure 5 are far more informative. Anyway, we added to the main text the results of nonparametric correlation analysis of RIs on age (L 539-542).

Figure 5B: Can the authors explain why they have plotted their reactivity index data on a linear scale? Given that it is a ratio I would expect to see it on a log scale.

The new S1 Fig shows RIs in log scale. We just wish to show there is no *plateau* in cord blood antibody levels (suggestive of IgG receptor saturation) as maternal antibody levels increase.

Figure 6: The image upload was of poor quality. It was difficult to discern the colours but his may be an upload issue.

Sorry! We now provide a figure file with much better resolution.

Table 2 – Please clarify which fifth is the highest level and what is the lowest level.

We added this information to Table 2. The reference is the group with lowest antibody levels. We added a similar information to the main text:

“On the contrary, we noted an increased risk of vivax malaria in **mothers with high antibody levels (quintile 5)**” (L 547-548)

L389-391: “associated with decreased malaria risk” – is this a typo? I believe the outcome in this section is anemia.

Many thanks! It was a typo! We changed to: “Maternal night blindness was significantly associated with decreased **anemia** risk” (L 592).

L389: Can the authors explain why maternal night blindness was considered as a risk factor for inclusion in the model?

Vitamin A, or retinol, deficiency is the most common cause of night blindness in the tropics and also a major cause of anemia. Although no association between vitamin A levels and anemia was observed in the mothers in our cohort (reference 32), we hypothesized that vitamin A deficiency in mothers might be a proxy of food insecurity in the household, leading to multiple micronutrient (including iron) deficiencies in the offspring.

Table 3 – Please five the units for age.

Done.

L444-451: An alternative explanation is that the API used in the model reflects an average API across an area that will not apply equally to all women and the women from an area with a given API who have experienced an infection during pregnancy are probably on average at greater risk of exposure and infection than those women from the same API area who did not experience an infection. Also worth noting that the model has assumed that the relationship between API and childhood malaria is linear.

The first comment refers to residual confounding. We agree with it and added the following sentence:

“We acknowledge some risk of residual confounding, since all mother-child pairs living in the same locality are assigned the same API but may still differ in malaria risk.” (L 691-693)

Regarding the second comment:

The expected value of the dependent variable is given in the models as a linear function of the predictor covariates, including API. This may not be the case of API, a key covariate for adjustment. To explore this, we did an additional analysis using APIs stratified into quintiles (first quintile, 0.409 to 2.652; second, 2.738 to 10.398; third, 13.574 to 126.395; fourth, 130.101 to 414.67; fifth, 435.74 to 1601.47). The models are described in S1 Text and results are shown in S1 Table. In conclusion, the main association observed, between maternal malaria in pregnancy and subsequent risk of malaria in children, remains unaltered when adjusting for the intensity of malaria transmission in the locality using API stratified into quintiles.

--------------------

**Summary and General Comments**

**Reviewer #1:**

This useful paper presents data collected as part of a large cohort study of mother-baby pairs in Amazonian Brazil. In this case, the focus of the analysis is morbidity from, and risk factors for, Plasmodium vivax malaria in children and mothers in the two years post delivery. Follow-up was primarily passive, relying on routinely-collected diagnostic data reported to the Brazilian Ministry of Health. A subset of over 300 remote-living mother-child pairs was excluded from many analyses as these individuals could not be reached for subsequent questionnaire administration and blood sampling at the two-year mark. A large proportion of mothers and children did not provide blood specimens at delivery. Despite these shortcomings, the analyses are robust and the results of significant interest. Salient findings include the relatively small number of children (7.1%) experiencing vivax malaria before their second birthday, the apparent protection against vivax malaria out to 1 year of age (substantially longer than passively transferred maternal antibodies could be expected to be present) and the marked concentration of cases in a small number of high-risk individuals. Repeated episodes of vivax malaria was associated with anaemia, something that has oft been postulated but not proved.

I have some fairly minor comments that I hope will be helpful for the authors.

1. Abstract, line 45: give a specific denominator here, not “>1,500 participants”

Corrected.

2. Abstract: the abstract essentially presents no methodology, other than to say the study was a “birth cohort”. At a minimum, the reader needs to know whether malaria detection was passive or active (passive in this case) and what means of diagnosis was used (ie microscopy, RDT, PCR). I would also suggest stating that a questionnaire was administered, and blood sampled at a two-year follow-up visit to mother and child.

We changed the abstract accordingly:

“Methodology/Principal findings

Overall, 1,539 children participating in a birth cohort study in the main transmission hotspot of Amazonian Brazil had a questionnaire administered, and blood sampled at the two-year follow-up visit. Only 7.1% of them experienced malaria confirmed by microscopy during their first 2 years of life – 89.1% of the infections were caused by *P. vivax*. Young infants appear to be little exposed to, or largely protected from infection, but children >12 months of age become as vulnerable to vivax malaria as their mothers. Few (1.4%) children experienced ≥4 infections during the 2-year follow-up, accounting for 43.4% of the overall malaria burden among study participants. Antenatal malaria diagnosed by microscopy during pregnancy or by PCR at delivery emerged as a significant correlate of subsequent risk of *P. vivax* infection in the offspring (incidence rate ratio, 2.58; *P* = 0.002), after adjusting for local transmission intensity. Anti-*P. vivax* antibodies measured at delivery do not protect mothers from subsequent malaria; whether maternal antibodies transferred to the fetus reduced early malaria risk in children remains undetermined. Finally, recent and repeated vivax malaria episodes in early childhood are associated with increased risk of anemia at the age of 2 years in this relatively low-endemicity setting.” (L 44-72)

3. Suggest use term “children under 5 years old” rather than “under-five children” to avoid ambiguity

Done.

4. Introduction: The salient results of the paper are presented in the last part of the introduction, before any of the methodology. This is very unorthodox and means the reader cannot appraise the validity of the results as they do not know how they were produced. I would suggest moving this result summary to the start of the discussion section.

Done. The last two sentences of the Introduction now read:

“Here we examine the burden of early life malaria in Juruá Valley, an Amazonian hotspot in northwestern Brazil that contributes nearly 18% of the country′s malaria cases [15]. We describe the incidence of *P. vivax* infection during the first two years of life in a prospective birth cohort study and its association with maternal antibodies and anemia at the age of two years” (L 146-150).

5. Methods: In the methods section, the authors state that serology was only done on 101 cord blood specimens. In the results section it becomes apparent that 637 cord blood samples were collected. Why the discrepancy? It would also be useful to document whether all mothers of the 6 neonates with congenital malaria were parasitaemic at delivery.

In this study, we tested a randomly chosen subsample of paired mother-cord blood samples for antimalarial antibodies (n = 101). We added the following explanation to Materials and Methods:

“To test whether maternal antibodies were efficiently transferred to infants, we compared antibody reactivity indices in a subset of 101 randomly selected paired maternal and cord-blood plasma samples from study participants. Importantly, maternal and cord blood antibody levels were strongly correlated to each other over the entire range of values (S1 Fig.), consistent with no saturation of the placental FcRn receptor [30] at high maternal IgG levels.” (L 301-313).

Note that, following reviewer # 3, this information was moved to Materials and Methods and Figure 5B is now S1 Fig and has been separated from Figure 5A (now Figure 5).

In a previous study (reference 21), we had tested all 637 cord blood samples available for malaria parasite DNA. The following paragraph from that publication provides the requested information:

“**Congenital malaria.** Cord blood samples from 637 neonates were tested for the parasite’s DNA. *Plasmodium vivax* and *P. falciparum* DNA was found in 4 (0.6%) and 2 (0.3% cord blood samples, respectively. These infants were born to mothers found to carry exactly the same parasite species at the time of delivery. Only one of these congenital infections became symptomatic over the next 4 weeks, being confirmed by microscopic analysis of peripheral blood and treated within 20 days after birth.” (From Reference 21, last paragraph of the Results section; freely available at: https://www.ajtmh.org/view/journals/tpmd/99/1/article-p73.xml).

We did not add this information to the present manuscript because there are results from a previous study, which have been published.

6. Methods, lines 234-236: Data on malaria episodes were collected passively and therefore cannot strictly be used to calculate prevalence. Suggest use the term “monthly incidence” (as per the excellent Figure 3).

You are absolutely correct! Changed to “monthly incidence” (L 322).

7. Methods: statistics generally well-described but there is no mention of the validity of the proportionality assumption for the Cox models. To my eye, the Kaplan-Meier curves suggest significant non-proportionality. How was this dealt with?

Thanks for pointing this out. The proportional-hazards assumption was tested on the basis of Schoenfeld residuals after fitting the final model using the *phtest* STATA command. We added this information to L 401-402. Indeed, the proportional-hazards assumption was violated by some 24-month survival models in mothers. We thus analyzed separately the periods between 0 and 12 months after delivery and the period after 12 months after delivery:

“The proportional-hazards assumption of Cox models was tested on the basis of Schoenfeld residuals after fitting the final model using the phtest STATA command. Because the proportional-hazards assumption was not fulfilled for mothers over the entire 24-month period, we carried out separate analyses for the periods between 0 and 12 months after delivery and the period after 12 years after delivery.” (L 416-421).

Using this approach, the proportional-hazards assumption was fulfilled.

8. Methods: there is no mention of antimalarial treatment protocols in the methods section – this only appears late in the discussion. In the methods I would suggest presenting the standard treatment protocol, at least for P. vivax, and providing details about eligibility criteria for primaquine. The relevant section in the discussion suggests to me that infants <6 months of age with documented normal G6PD activity are prescribed primaquine (outside of WHO guidelines but quite possibly the right thing to do). Is this correct?

We added the following information:

“*Plasmodium vivax* infections were treated with 25 mg/kg of body weight of chloroquine administered orally over three consecutive days and 3.5 mg/kg of primaquine administered over 7 days. Primaquine was omitted in children over 6 months of age, as recommended by the Ministry of Health of Brazil at the time of the study (2015-2018) [25].” (L 260-263).

Reference 25 (malaria treatment guidelines of the Ministry of Health of Brazil) has been added. The following text (bold) was added to Discussion:

“Although primaquine is routinely prescribed with chloroquine to suppress *P. vivax* relapses in Latin America [1], infants <6 months of age with unknown glucose-6-phosphate dehydrogenase (G6PD) deficiency status are considered primaquine-ineligible in Brazil because of the risk of hemolysis [25]. **Because G6PD testing is not widely available, young infants are very rarely prescribed primaquine.** Moreover, not all eligible children and adults adhere to the unsupervised 7-day primaquine treatment [52] and shortened anti-relapse regimens are urgently needed [52].” (L 753-759)

9. Results and discussion: I would caution against implying causality when referring to the association between repeat vivax malaria events and subsequent anaemia. Although there is biological plausibility for this, there are many other confounders that may be relevant.

We agree. We changed the text accordingly (L 736-737).

10. Results: The authors lapse into speculation in several places in the results section. I would present the facts in the results and leave the speculation to the discussion.

We removed all discussions from the Results section.

11. Conclusion (both abstract and main paper): I think the conclusion that repeated infections and subsequent anaemia impede normal growth and development cannot be claimed on the basis of the results of the current study. I would suggest removing the phrase “which impedes their normal growth and development”.

We agree and have removed this phrase. We meant that anemia (which is associated with repeated infections in this population) has well-known consequences on child development, but do not provide new evidence for that.

12. Figure 4: Perhaps an editorial problem, but figure 4 seemed to be very low resolution and I could not read the text.

Sorry! We uploaded a file with higher resolution.

**Reviewer #2:**

Summary

Pincelli et al have conducted an interesting study on a prospective birth-cohort in Brazil. Specifically the focus of this paper is on factors associated with malaria in children over two years; malaria in mothers over two years and anemia in children at two years. A particular focus is on the exposure of P. vivax antibodies as an exposure variable. I congratulate the authors on putting together this impressive piece of work. The data presented within this paper are valuable for the community. My primary concerns are that the authors place a little too much confidence in their ability to account for confounding. Studies seeking to relate malaria-specific antibodies to malaria related outcomes will always face difficulty accounting for the confounding of overall malaria and vector exposure. As such I think the authors need to be a little bit more circumspect in their conclusions and acknowledge the possibility of residual confounding explaining some of their results.

**General comments**

The authors use unadjusted bivariable analysis to screen risk factors for use in multivariable analysis and have only retained covariates which have associations with the outcome at a significance level of 10%. There needs to be some discussion of the shortcomings of this approach. I recommend the following articles: https://www.sciencedirect.com/science/article/pii/089543569600025X

https://doi.org/10.2105/AJPH.79.3.340

https://journalofbigdata.springeropen.com/articles/10.1186/s40537-018-0143-6

We completely agree with the above criticisms regarding standard stepwise selection. We introduced a few changes to explain our variable selection strategy, which is clearly different from the one criticized in the articles above:

“Variables associated with the outcome at a significance level <20% in unadjusted analysis were further assessed in negative binomial models.” (L 347-349).

Note that the first step used a very relaxed criterion **(*P* < 20%)** for inclusion in adjusted models. In other words, we did not use a significance level of 10% in unadjusted analysis to select covariates for inclusion in multivariable analysis.

The next step of variable selection is now described as follows:

“We next used a hierarchical approach based on conceptual frameworks with distal, intermediate and proximal levels of disease determination (S2 Fig.) [35], to select covariates that were retained in the final adjusted models. At each level of determination, covariates were retained in downstream analyses if they were associated with the outcome at a significance level of <10% or if their inclusion in the model changed the risk measures by >=10%.” (L 349-353).

Note that this approach is entirely different from that criticized in the literature in many ways. First, we “do not throw all explanatory factors in the model and see what turns up as statistically significant” using stepwise regression. (We quote from reference 35, the classical 1997 paper by Victora et al. freely available at: https://academic.oup.com/ije/article/26/1/224/730584.)

Instead, we build a hierarchical model of disease determination and use a stricter statistical criterion (*P* < 0.10) to select variables within each hierarchical level. Once selected, these variables are maintained in the final model, after adding variables corresponding to more proximal levels of determination, irrespective of *P* values after adding these variables.

Note that we do not treat all variables as if belonging to the same level of disease determination, but follow a prespecified hierarchical model shown in S2 Fig (malaria) and S3 Fig (anemia). Importantly, some covariates may be retained in the model (e.g., demographic variables) even if they are not statistically associated with the outcome but may modify other associations.

“Malaria in pregnancy emerged as a significant correlate of subsequent risk of malaria in the offspring, which is not prevented by maternal anti-P. vivax antibodies transferred to the fetus.”

I think the conclusion that maternal anti-P. vivax antibodies are not preventing any detected malaria in the offspring is not warranted given the data. My two main reasons for this belief are:

1. Given the extremely strong relationship between levels of maternal antibodies and maternal malaria I am scheptical that there is no residual confounding of malaria exposure risk for the mother (and child).

2. Even if the model had perfectly accounted for all confounders (or if these estimates were from an RCT) then I would not conclude that there is no association. The lower bounds of the 95% CI for each vivax antibody upper fifth are 0.4, 0.5 and 0.4 so it would be inappropriate to rule out a protective effect, simply put the uncertainty around the estimates is large and the data are compatible with clinically relevant associations in both direction - see https://www.bmj.com/content/311/7003/485.full

We agree with these comments and introduced a few critical changes into the text. First, we changed the abstract:

“Anti-*P. vivax* antibodies measured at delivery do not protect mothers from subsequent malaria; whether maternal antibodies transferred to the fetus reduced early malaria risk in children remains undetermined.” (L 54-70).

Next, we changed the Results section accordingly:

“Elevated reactivity indices of specific antibodies at delivery were not associated with protection from vivax malaria **among mothers** (Fig. 6A). On the contrary, we noted an increased risk of vivax malaria in mothers with high antibody levels (quintile 5). Antibody levels were significantly and positively associated with the HR for the time to the first vivax malaria in mothers up to 12 months following delivery, after adjusting for potential confounders (Table 2). Not surprisingly, however, the association between antibody levels and maternal malaria risk shortly after delivery was greatly attenuated over the second year of follow-up (Table 2), as antibodies appear essentially to indicate recent malaria exposure but not future long-term exposure or risk. Because vivax malaria was infrequent among study children before the age of 1 year, with only 14 children with complete serological data suffering one or more infections, the association between maternal antibodies and early life malaria could not be properly assessed (Fig. 6B). **Cox regression models showed no significant association between maternal antibody levels and risk of malaria during the first two years of life** (Fig. 6B and Table 2).” (L 546-552)

We therefore state that:

1. Maternal antibodies are significantly associated with increased malaria risk in mothers.

2. Malaria among children during their first year of life was too infrequent for a proper analysis of its association with maternal antibodies.

3. When extending the analysis to the first two years of life, we found no evidence of association between maternal antibodies and malaria in children. We note that this is not surprising, given that the putative protective effect of maternal antibodies is not expected to last > 6-8 months in the offspring.

Finally, we added to the discussion:

“Maternal antibodies have been hypothesized but not conclusively shown to reduce the risk of P. vivax infection in young infants [5]. One may argue that passively transferred immunity is unlikely to work in the infant while the mother continues to suffer from malaria, as we have observed in our cohort participants (Fig. 3). However, conventional serology detects both protective and nonprotective antibodies and high antibody levels may indicate increased exposure rather than reduced risk [38]. The fine specificity and functional properties of parasite-inhibitory anti-P. vivax antibody responses remain largely unexplored [38,39]. The positive association between antibodies measured at delivery and subsequent malaria risk in mothers was greatly attenuated over the second year of follow-up (Table 2), suggesting that antibodies essentially indicate recent malaria exposure, but not future long-term exposure or risk. Antibody concentration may also matter for infant protection. Levels of parasite-inhibitory antibodies that fail to protect heavily exposed mothers may suffice to protect their less exposed offspring if placental transfer to the fetus during pregnancy is efficient.” (L 656-668)

This is a critical point. Given the rarity of malaria in young infants, we cannot test directly if passively transferred maternal antibodies can protect them from malaria. However, we were able to test whether these antibodies appeared to protect mothers from malaria. This analysis has rarely been done in the literature of maternal antibodies and malaria. We next discuss some hypotheses regarding antibody-mediated protection from vivax malaria.

The authors could consider modelling haemoglobin levels directly as a supplementary analysis, rather than dichotomising into anaemia yes/no as this would improve power.

Thanks for suggesting this. Unfortunately, hemoglobin concentrations are not normally distributed in our study children (Shapiro-Wilk test for normality, *P* <0.001). We thus run a quantile regression analysis to explore associated factors for hemoglobin values at each point of the 25th, 50th and 75th percentiles. “Quantile regression was used to further explore the association between malaria and hemoglobin levels at the age of 2 years at the 25th, 50th, and 75th quantiles, adjusted for the covariates shown in S2 Fig.” (L 466-473).

The results are presented in S2 Table and summarized in the main text. “Quantile regression analysis revealed a significant association between repeated vivax malaria episodes and decreased hemoglobin were significant for the 25th percentile (-5.10 g/L [95% CI, -9.71 to -0.50 g/L]) and the 50th percentile of hemoglobin values (-6.35 g/L (95% CI, -12.44 to -0.26 g/L), while adjusting for child age and sex, wealth index, whether the mother is beneficiary of the Bolsa Família program (no vs. yes), maternal schooling, whether the mother is the household head, household size, maternal anemia at delivery, and gestational age (S2 Table). “ (L 607-613).

--------------------

**Reviewer 3:**

Overall comment

The paper summarises findings of birth cohort in a malaria-endemic setting in the Brazilian

Amazon. The paper contains a lot of interesting data on P. vivax infection that is very much

worth publication. However, the current format is relatively difficult to digest. This is

because the paper focusses on a number of outcomes (not all of them are immediately

clear), and uses a wide range of analytical approaches. It effectively reads like a combination

of three small somewhat related papers merged into one. It reports on the burden of

postpartum clinical malaria in the mother, the burden of clinical malaria in the first two

years of life of the child, the association between gestational malarial infection (?PCR) and

clinical malaria in the child, the relationship between P. vivax infections in the first two years

of life and anaemia at Year 2, amongst others.

I recommend to focus the story of the paper on P. vivax infections during the first 2 years of

life (90% of infections were vivax infections), and factors associated with these infections, in

particular maternal P. vivax status. Less is more when it comes to telling a consistent and

interesting story, which this is. Telling the story would be strengthened by more clearly

highlighting how malaria during pregnancy was diagnosed (and species breakdown), and

restricting analyses to mother -infant pairs for whom you were able to determine the API,

maternal infection status, and clinical malaria follow-up for 2 years in children.

We have tried to emphasize some main topics in the paper. First, we provided additional information regarding the diagnosis of malaria in pregnancy in our cohort:

“The diagnosis of malaria in pregnancy was defined by combining antenatal diagnosis by microscopy of Giemsa-stained capillary blood thick smears prepared during sick visits to public clinics and species-specific real-time PCR [20] carried out on venous blood samples collected at delivery [21]. “ (L 203-206). Reference 21 is freely available at: https://www.ajtmh.org/view/journals/tpmd/99/1/article-p73.xml.

The analysis of risk factors for malaria in children is limited to those with API information and attended the 2-years follow-up visit. All of them (n = 1,494) had information regarding maternal malaria in pregnancy (please see Table 1).

Relationships between levels of three anti Pv antibodies measured in maternal blood and P.v. in first two years of life could be integrated into the story, but the story of anaemia is probably more relevant.

An important finding of this study is that malaria is very infrequent during the first year of life. To explore these findings: (a) we tested whether malaria was similarly infrequent among mothers who share the same household with their children and (b) we tested whether maternal antibodies passively transferred to the fetus might explain the reduced risk of malaria during the first year of life.

The second analysis is severely limited by our lack of statistical power. However, we show that maternal antibodies do not appear to protect mothers from malaria. We therefore discuss whether such antibodies, which do not protect mothers, are likely to protect their offspring.

We hope that these relationships are now clearly stated.

In addition to API, adjusting analyses of the association between gestational Pv with infant Pv for maternal postpartum clinical vivax episode may help to deliver some further insights regarding the mechanisms of the aforementioned association.

Malaria incidence in mothers and APIs are strongly correlated to each other. Because of collinearity, we must choose one of them as a marker of exposure in children. API is clearly a better marker because – as acknowledged by the reviewer – malaria in mothers include both new infections (= exposure shared by mother-child pairs) and relapses, since primaquine was not administered during pregnancy (risk of relapse much higher in mothers). We therefore chose IPA as a proxy of malaria exposure.

There is a parallel story of maternal postpartum malaria, and the relationship between

levels of three anti Pv antibodies measured in maternal blood at birth and maternal

postpartum malaria and infant malaria. This complicates the paper and would probably be

best told as a stand-alone paper or in a supplemental file, highlighting how pre and

postpostum infection risk compares in mothers.

As above. We examined whether maternal antibodies, which are often mentioned as a potential protective factor against malaria in infants, were actually able to protect mothers from malaria. This is a clear knowledge gap.

However, we agree that a complete analysis of how antenatal malaria risk compares with post-partum risk – a controversial topic in the *P. falciparum* literature – must be left for a future stand-alone paper.

Figure 3 A is helpful though in a way. The main issue with these data to is the lack of regular PCR-based screening for infection during and after pregnancy, which precludes a conclusive analysis of P. vivax risk in pregnant versus postpartum women. The measured antibodies are more likely to represent a marker of current or recent infection in others (and thus a predictor of relapse in mothers).

Figure 3 allows us to tested whether malaria was similarly infrequent among mothers who share the same household with their infants. The clear answer is no, suggesting that children are somehow “protected” while their mothers are not. We discuss some hypotheses to explain the differences observed. We think that the comparison between malaria incidence rates in children and their mothers is one of the critical aspects of this manuscript, and reviewer #1 appears to agree when he mentions the “excellent Figure 3”.

Include a STROBE guideline tick chart as a supplementary file and ensure the paper is

formatted accordingly.

Done!

Include a Table (new Table 1) summarising the main characteristics of your cohort.

We have not included such a table because this cohort has been described in a recent “cohort profile” article published in an open-access journal (Tables 1 and 2 of reference 16), but Figure 2 (flow chart) provides the relevant information regarding study populations and sample sizes.

Ensure that your malaria measures are clearly explained (differentiate between infection

and clinical malaria).

Done! In fact, all laboratory-diagnosed vivax malaria episodes retrieved from the case notification database were “clinical” (i.e., symptomatic) malaria episodes. We added the adjective “clinical” accordingly.

Rationalise the outcome measures for your study and how they were assessed – there are a

great range of them, ORs, IRR, cumulative incidence, HRs etc etc. – the are all reasonable

but need to be presented in a more coherent manner.

Done. Please see below.

**Abstract**

Overall comment:

The methodology section needs to be strengthened, e.g. how and when was malaria

assessed, details of antibody assays and time points, surveillance frequency etc.

We agree and changed the abstract accordingly:

“1,539 children participating in a birth cohort study in the main transmission hotspot of Amazonian Brazil had a questionnaire administered, and blood sampled at the two-year follow-up visit. Only 7.1% of them experienced malaria confirmed by microscopy during their first 2 years of life – 89.1% of the infections were caused by P. vivax. Young infants appear to be little exposed to, or largely protected from infection, but children >12 months of age become as vulnerable to vivax malaria as their mothers. Few (1.4%) children experienced >=4 infections during the 2-year follow-up, accounting for 43.4% of the overall malaria burden among study participants. Antenatal malaria diagnosed by microscopy during pregnancy or by PCR at delivery emerged as a significant correlate of subsequent risk of infection in the offspring, after adjusting for local transmission intensity. Anti-P. vivax antibodies measured at delivery do not protect mothers from subsequent malaria; whether maternal antibodies transferred to the fetus reduced early malaria risk in children remains undetermined. Finally, recent and repeated vivax malaria episodes in early childhood are associated with increased risk of anemia at the age of 2 years in this relatively low-endemicity setting.” (L 44-68)

The background section could include a mention of the lack of knowledge around the

relationship between P. vivax malaria in pregnancy and risk of P. vivax infection in infants.

We agree that this is a key knowledge gap and discuss this topic in the main text, but were unable to find room for this in the abstract, given the strict word count limits.

“Malaria causes significant morbidity and mortality in under-five children in sub-Saharan

Africa and the Asia-Pacific region, but neonates and young infants remain relatively

protected from clinical disease putatively due to the transplacental transfer of maternal

antibodies.”

Comment: Suggest to make two sentences out of this to improve readability

Thanks for the suggestion! Done.

“Plasmodium vivax malaria in early childhood – traditionally viewed as a benign infection…”

Comment: Are you referring to the more global statement of vivax being a ‘benign’ malaria,

or specifically to P. vivax infections in early childhood being viewed as benign – pls clarify.

Both. There is an extensive literature about *P. falciparum* malaria in under-five children – not surprising, given its immense contribution to infant morbidity and mortality in sub-Saharan Africa. However, very little has been published on vivax malaria in early childhood in the Americas. A series of key papers have conclusively shown that vivax malaria causes substantial infant morbidity and mortality in the Asia-Pacific region, but these results appear to be ignored elsewhere. Importantly, IPTp strategies are currently not recommended for vivax malaria.

“We show that only 7.1% of the >1,500 participants in a birth cohort study...”

Comment: clarify participants = children; 7.1% (n=?);1,500 – put precise number here.

Done.

Line 50-51 How was malaria in pregnancy defined

Done:

“The diagnosis of malaria in pregnancy was defined by combining antenatal diagnosis by microscopy of Giemsa-stained capillary blood thick smears prepared during sick visits to public clinics and species-specific real-time PCR [20] carried out on venous blood samples collected at delivery [21].” (L 203-206).

Line 48 children rather than toddlers

Changed.

Line 49 – few children (1.4%)

Changed.

Line 50-51 – put some stats here to support your argument. State that you adjusted for

the transmission setting shared by mother and child – this is good!

Done.

Line 52 present your antibody findings in a separate statements – levels of these three

antibodies did not associate with risk of P. vivax at 2 years. Not enough infections in

first year of life to clearly state if they had an impact there.

Done.

Line 57 – The conclusion read like a conclusion for this paper in combination of the

previously paper published paper by the same group. It should instead focus on

findings from this study only.

Changed accordingly.

Line 59 – Maternal infection, the majority being P. vivax, associated with an increased risk of infants getting treated for clinical P. vivax malaria to local health centres. Then add your thoughts on what this means for prevention of malaria in pregnancy and infancy in your setting.

We added to the Conclusions section a comment on the implications of our findings for prevention of malaria in pregnancy and infancy in the Amazon.

Introduction

Overall comment:

I would remove mention of the ‘results’ in the last paragraph of this background section.

Done.

I would include more information on research that has assessed relationships between malaria

in pregnancy and malaria in infants, including their methodological shortcomings to adjust

for shared exposure risk. Highlight the lack of P. vivax studies on this subject.

Done: “The adverse health effects of vivax malaria in the course of the first 1000 days – from conception to child’s second birthday – remain understudied in the Americas [14]. A critical knowledge gap refers to the association between maternal malaria in pregnancy and risk of subsequent P. vivax infection in the offspring. No population-based data are available for Brazil, a country that contributes 20% of malaria cases on this continent [2].”(L 141-144). Further comments on shared exposure are made in the Discussion.

If available, I would also mention any studies that looked at maternal P. vivax antibodies and any protective effects for the offspring, should you decide to keep this.

We found no population-based study worth mentioning in this context.

Line 95-96 and appears to be more resilient than P. falciparum to current control

Comment: Do the authors want to comment as to why they think this is? I think it matters!

Added.

Line 108

Comment: I would state that P. vivax is the most widespread malaria parasite globally:

https://www.ncbi.nlm.nih.gov/pmc/articles/PMC5198891/

Is this correct geographically? The historical distribution of vivax malaria is surely more extensive than that of *P. falciparum*, but does this hold now? Most areas where eradication was achieved in the 1950s and 1960s were Pv-dominated areas. More recently, malaria elimination was again successful in vivax-dominated territories (e.g. Argentina and Paraguay, the Middle-East ). The vivax malaria map has shrunk much more than the *P. falciparum* distribution…

Line 117: which continent?

Rephrased to make it clearer: “No population-based data are available for Brazil, a country that contributes 20% of malaria cases in Latin America and the Caribbean region [2].”

Line 117-119

Comment: Clarify that the scope of the study. There are many aims – characterize the burden

of malaria in the first two years of (ex-utero) life; assess relationship between MIP and risk of

infant malaria; assess relationship between maternal anti-P. vivax antibodies and risk of

infection in infants; association of P. vivax and anaemia at age 2

Done: “We describe the incidence of P. vivax infection during the first two years of life in a prospective birth cohort study and its association with maternal antibodies and anemia at the age of two years.” (L 148-150).

**Materials and Methods**

Overall comments:

It would be good to state more how each statistical approach addresses a particular aim of the

study. It was challenging to work out what the different analyses were addressing. The core

of this is to clearly address and refine the aims first (see above).

Done. Before describing each of the three multivariate models we now describe the question to be addressed: (a) “We used negative binomial regression models **to identify correlates of malaria risk among children**”; (b) “We used survival analysis **to test whether levels of specific maternal antibodies measured at delivery predicted vivax malaria risk in mothers and in their children over the next months**.”, and (c) “We next used multiple logistic regression analysis **to test whether previous malaria episodes in children were associated with an increased risk of anemia at the age of 2 years** (dichotomic outcome variable)”.

Please provide some background details on malaria prevention and detection strategies during

pregnancy, as well as after pregnancy, in your study settings. What is the relapse timing and

frequency of P. vivax in your setting? How does it compare to other settings?

We added the following: “Once infected with *P. vivax*, 23% of the pregnant women in this setting are estimated to have one or more vivax malaria recurrences over the next 12 weeks. The vast majority (86%) of these early *P. vivax* recurrences are likely relapses [22].” (L 206-209)

The description of covariates is lengthy and complicated, especially as you are using different

covariates for your analyses. This could be rationalized.

We moved this description to S1 Text.

Line 137 – Mother-baby pairs were enrolled during pregnancy at public antenatal clinics, or

at birth etc…

Changed.

146 – API, number of laboratory-confirmed clinical malaria cases.

Changed

Line 150 – this link should go into the reference section. How does this infant mortality

compare to the rest of the country.

Added (L 190-193). We left the link here because we do not cite formal publications but rather datasets (as we did in previous publications in PLoS NTD), but may move it to the references at the editor’s discretion.

Line 158-160 – Please clarify: a) PCR assay used; PCR was done for all mothers; c) PCR

was done on which blood – maternal peripheral, placental etc - you cannot expect the reader

to go through your previous publications.

Done. “The diagnosis of malaria in pregnancy was defined by combining antenatal diagnosis by microscopy of Giemsa-stained capillary blood thick smears prepared during sick visits to public clinics and species-specific real-time PCR [20] carried out on venous blood samples collected at delivery [21].” (L 203-206).

Line 161 – clarify make of baby scale – you subsequently never mention birthweight again so

wondered whether a) birthweight should be included in your analyses as it may affect malaria

risk – if you decide not too you can as well remove this bit.

Clarified. Birthweight is one of the covariates included in malaria and anemia risk models (please see S2 and S3 Figs and S1 Text).

Line 162-164 – reword this so it’s a bit clearer what happened to the rural women/infants.

Done. “Study participants were invited to attend a follow-up assessment at the age of 2 years, except for those living in remote rural communities (n = 302), typically with no electricity and cell phone coverage, who were considered ineligible because they could not be reached by telephone or social media.” (L 211-214)

176 – 178 – can you confirm if these data refer to cases in symptomatic infants/women –

that’s how I understand it so perhaps add ‘computed microscopy-confirmed clinical malaria

episodes… etc

Yes, symptomatic malaria episodes. Changed accordingly.

211 – 213 – Can you clarify here that you did not lump these together for your analyses but

looked at correlation between maternal and infant levels (this only becomes clear in the

results section)

We changed the text and moved the comparison between maternal and cord blood antibody levels to the new S1 Fig.

234 – 236 is it the cumulative incidence (birth to age 2) that you were calculating?

Sorry, our mistake. We calculated monthly incidences. Corrected.

238 – please clarify who these participants are – kids or mums. Do this throughout the

manuscript.

Done.

241 – if you make infant malaria risk a main outcome of your study, then it might be easiest

and clearest to restrict all analyses to this group (i.e. mother infant pairs for whom you can

establish an API).

This is precisely what we did; adjusted analyses of malaria risk are restricted to children with API information (n = 1,494).

241 – 244 you state here that regression models were built for continuous outcomes total

number of lab confirmed malaria episodes, and lab confirmed vivax episodes. I have

struggled to subsequently find these analyses in the manuscript. Do you mean here that you

calculated IRR of any clinical malaria, or P. vivax malaria, as the ratio of clinical malaria

episodes in children per person-year by maternal infection. Could be a lot clearer

We did separate analyses for two outcomes in children: (a) any clinical malaria (Pv, Pf and mixed-species infections included) and (b) vivax-only malaria episodes.

We used IRRs (also known as incidence density ratios) as usual in cohort studies (e.g., “rate ratios” at https://www.cdc.gov/csels/dsepd/ss1978/lesson3/section5.html). We compared malaria incidence rates in subjects that differ according to key exposure variables (incidence rate in exposed/incidence rate in unexposed) while adjusting for potential confounders. For example: incidence of malaria (any malaria or vivax malaria) in children exposed to malaria in utero/incidence of malaria (any malaria or vivax malaria) in children not exposed to malaria in utero.

To simplify the model description, we moved the list of covariates to the S1 Text.

245 –Would maternal smoking status be relevant? Sex of the infant has been reported to

modify malaria risk so that may also need to be considered. Bed net use is also key but not

reported – discuss this in your limitations section. I mentioned birthweight earlier.

We are not aware of any reason why maternal smoking and infant’s sex should be relevant for the malaria risk analysis. The availability and use of insecticide-treated bednets has not been investigated during the follow-up visit. We do have data on overall bednet use but we do not distinguish between those that are insecticide-treated from regular bednets.

Importantly, use of insecticide-treated bednets in this region is a proxy of exposure (collinear with API), rather than a correlate of protection (see, e.g., reference 41). This is because insecticide-treated bednets are selectively distributed in areas with the highest APIs, but not in those with limited transmission. Regular (not insecticide-treated) bednets are widely available, but confer relatively little protection against malaria.

Moreover, because of the early-evening and outdoor-biting behavior of local malaria vectors, insecticide-treated bednets tend to be much less effective in the Amazon compared with endemic settings in Africa (see, e.g., references 1 and 15 for a discussion).

252 – is API normally distributed?

No (Shapiro-Wilk test, z = 13.455, *P* < 0.000001).

We did an additional negative binomial regression analysis using average APIs stratified into quintiles (first quintile, 0.409 to 2.652; second, 2.738 to 10.398; third, 13.574 to 126.395; fourth, 130.101 to 414.67; fifth, 435.74 to 1601.47). As shown in S1 Table, covariates associated with malaria in young children remain the same after adjusting for API stratified into quintiles.

269 – is this IRR calculated from a ratio of the number of episodes per person-year by

maternal gestational malaria status? Clarify the denominator

The numerator is the number of malaria episodes experienced by each child during their first two years of life. The denominator is 2,988 person-years. Given that each of the 1494 children were followed up for two years (i.e., we retrieved case notification records for each child from birth through the second birthday), each child has contributed exactly two person-years of follow-up. We added this information to the text:

“We used negative binomial regression models to identify correlates of malaria risk among children. This analysis comprised all children assessed at delivery, except for those with unavailable API estimates for their areas of residence (n = 45). This gives a total of 1,494 children for whom malaria morbidity data from birth to their second birthday were retrieved from the case notification database, or 2,988 person-years of follow-up.” (L 326-330).

273 – I would focus on infants. Introducing the story the association between anti-Pv

antibodies in the mother at birth and protection from subsequent infection in the other is

interesting but diverts away the attention from the core part – impact on infection risk in

infants.

Please see above: “An important finding of this study is that malaria is very infrequent during the first year of life. To explore these findings: (a) we tested whether malaria was similarly infrequent among mothers who share the same household with their children and (b) we tested whether maternal antibodies passively transferred to the fetus might explain the reduced risk of malaria during the first year of life.”

Postpartum infections in the mother could be used as a covariate in your multivariable analyses, as a second marker of shared exposure risk (and for vivax, relapses in the mother may be the primary course of infection in their kids).

Malaria incidence in mothers and APIs are strongly correlated to each other. Because of collinearity, we must choose one of them as a marker of exposure in children. API is clearly a better marker because – as acknowledged by the reviewer – malaria in mothers include new infections (= shared exposure) and relapses (since primaquine was not administered during pregnancy). We therefore chose IPA as a proxy of malaria exposure.

281 – previous malaria episodes – in the child?

Yes. Changed accordingly.

283 – when you say ‘participants’ spell out which participants you mean – children or

mothers

Done.

285 – why include the child’s age in days as a covariate at the 2 year follow – the child

should be 2 years old.

Thanks for raising this point. We scheduled four follow-up visits during the second year of study. Children were scheduled to follow-up visit at the time they were 2 years ± 3 months old (average, 725.87 days; standard deviation, 44.46; range, 488 to 980 days). Because some children missed the appointments or their mothers requested an earlier appointment, there was some variation in age at the time of hemoglobin measurement that must be accounted for in the adjusted models. We added the following explanation:

“This analysis comprised 860 children who had hemoglobin measurements at the age of approximately 2 years (average, 725.9 days; standard deviation, 44.5 days). Covariates included in the multiple logistic regression models and the hierarchical model for variable selection are shown in S3 Fig.; all models controlled for age to account for individual age variation, since children were not exactly 2 years old.” (L 428-432).

Note, however, that data on malaria episodes experienced by each child were obtained for exactly 2 years – from birth to the second birthday. For this reason, the follow-up duration is exactly two years, even if the child was not exactly 2 years old at the time of the “2-year” follow-up visit.

295 – 303 – clarify that this is malaria in the infant/child, and that these categories were used

to determine relationships between malaria infection in children.

Done.

**Results**

311-312 – are you trying to say that all these episodes were ‘uncomplicated disease episodes

(defined how)’?

We added: “Only uncomplicated clinical disease was observed and treated on an ambulatory basis; **no child was hospitalized due to malaria**.” (L 483-484).

312 – 314 – I would remove the maternal data here and focus on infant malaria story first.

As above. The comparison between malaria incidence in children and their mothers is a critical part of the results.

316 – first sentence – remove – this is stuff for the discussion.

Done.

342 – Malaria in pregnancy – can you spell out in your results section how many women

were infected at birth (it’s in the table but needs to be moved to the text), and the main

species of maternal infection at birth.

We added the following information: “Malaria in pregnancy, **diagnosed in 178 (11.9%) mothers [21]**, was significantly associated with subsequent malaria risk even after controlling for transmission intensity in the children′s area of residence”. We note, however, that this is published information. Overall, 74.6% of malaria episodes diagnosed during pregnancy by microscopy and 83.1% of those diagnosed at birth by PCR were to *P. vivax* (reference 21).

343 – 348 this would probably fit in the discussion section. Instead, it would be interesting to

see the data on maternal postpartum infection and its relationship with infections in children.

Done; moved to Discussion.

358 – 362 – this could almost be put in the methods. It attempts to validate the notion that

maternal levels reflect cord blood (infant) antibody levels (levels are usually higher in cord

blood compared to mat plasma), which then helps you to comment on the relationship

between maternal antibody levels and risk of infection in infants.

Done; moved to Methods (L 301-313) and S1 Fig (= former Figure 5B).

367 – we noted

367-368 we noted an increased risk of postpartum clinical vivax malaria in mothers with high

antibody levels (quintile 5).

Corrected, thanks!

371- 373 – for discussion – just present the data here.

Done: moved to Discussion: “The positive association between antibodies measured at delivery and subsequent malaria risk in mothers disappeared over the second year of follow-up (Table 2), suggesting that antibodies essentially indicate recent malaria exposure, but not future long-term malaria exposure or risk. Antibody concentration may also matter for infant protection. Levels of parasite-inhibitory antibodies that fail to protect heavily exposed mothers may suffice to protect their less exposed offspring if placental transfer to the fetus during pregnancy is efficient.” (L 663-669).

366 – 376 – please mention the fact that anti Pv Ab levels are not associated with infant

malaria (DBP maybe?). All you mention here is that you have not done this analysis for

infants, and we have to dig in the table to find out what happened to the children.

Done (L 577-578).

384-385 – define anaemia in the methods section. What are your exposures, what are you

outcomes – this needs to be clearly stated in the methods.

Done (L 237-238 and again in Results (L 586).

387-388 define Bolsa family as a poverty indicated in methods, and then use poverty

throughout in your results.

Bolsa Família **is not** synonymous with poverty. All Bolsa Família recipients are poor, but importantly, not all poor families participate in the Bolsa Família program. There is an extensive literature showing that Bolsa Família recipients have significantly better health indicators than non-recipients with comparable socioeconomic status. The poorest of the poor have no access to Bolsa Família. That is why we included both Bolsa Família and wealth index among our socioeconomic covariates. Please note that the final models shown in Table 1 include both variables.

Ref to your Supp Fig 2 here for the reader to be reminder what covariates you looked at. Done, L. 589). Would maternal gestational malaria be relevant? Not directly (see S3 Fig.). However, because malaria in pregnancy is positively associated with malaria risk in the offspring, which in turn is associated with increased risk of anemia, there is an indirect relationship.

**Discussion**

Overall comment:

I would summarise the main findings of the overall study in the first paragraph, and then

move on to discuss the individual points/discoveries.

Done.

Line 417 – here we show that clinical vivax malaria etc…

Changed.

418 and following – how do you explain your findings – you are only suggesting that the low

incidence of P. vivax malaria in the first year of life may not be entirely mediated by maternal

antibodies or fetal haemoglobin, the protective ability of which you suggest weans after the

first six months. The references used in this paragraph largely related to falciparum infections.

“We hypothesize that infants <12 months are infrequently infected because they remain largely unexposed to vectors. First, they are little affected by outdoor malaria transmission, which predominates over indoor transmission in most of the Amazon [15], until they start to walk and actively explore the external environment. Second, infants may be less attractive to mosquito vectors than older children [40], partially due to their smaller body size. Third, long-lasting insecticide-treated bed nets, which are available in high-transmission localities across the study site [41], are likely to avert more infectious bites among infants who typically sleep >12 hours per day, compared with older children.” (L 671-678).

424-431 Explain the findings of your study in the context of the published data of P. vivax

antibody studies and protection from infection. Focusing on research that investigates the role

of passive immunity for Pv would be most interesting. Are your antibody targets markers of

maternal infection (including latent infections) rather than those components of the immune

system that are associated with protection from infection? They associated with recurrence in

the mother, probably due to relapse.

“However, conventional serology detects both protective and nonprotective antibodies and high antibody levels may indicate increased exposure rather than reduced risk [38]. The fine specificity and functional properties of parasite-inhibitory anti-P. vivax antibody responses remain largely unexplored [38,39]. The positive association between antibodies measured at delivery and subsequent malaria risk in mothers disappeared over the second year of follow-up (Table 2), suggesting that antibodies essentially indicate recent malaria exposure, but not future long-term exposure or risk.” (L 660-666).

433 – 440 this would fit with the paragraph staring at line 418

We first explain why antibodies are unlikely to account for the reduced risk of malaria in infants and next offer some hypotheses to explain this finding.

442 – 453 This to me is one of the key findings of the study, and the big novelty, as you are

showing it for P. vivax. I would expand this analysis to see if this association between

malaria during pregnancy and malaria in the child remains if maternal postpartum malaria

was included in the model.

Please see above. We argue that maternal malaria incidence and APIs are colinear (we cannot keep both in the models) and API more precisely reflects malaria exposure in children.

460 – we note that hb measurements were taken in &gt;70% of the children in our cohort at the 1 year etc… and X % of children were anaemic and thus supplemented.

Added as suggested: “Although we found much less anemia among MINA-Brazil cohort participants at the age of 2 years (prevalence, 13%), compared with proximate sites in the Amazon [46,47], we note that hemoglobin measurements were made in 76.6% of the study participants at the 1-year follow-up visit [16] and those with anemia (41.0% [95% CI, 37.3 to 44.8%]) were prescribed iron supplements. Treatment was unsupervised, but most anemic children may have had a sustained hemoglobin response following oral iron administration.” (L 707-722).

465 – merge this paragraph with the subsequent one, and abbreviate things somewhat.

We first provide information on the prevalence of childhood anemia in the study area and next focus on anemia in vivax malaria (one of the main topics of the paper). The association between vivax malaria and anemia is a key finding of this article – we cannot see how to abbreviate this discussion.

480 – highlighting the biology of P vivax in your introduction would be good, as it shapes our

thinking around the origin of blood stage infections in the mother, and the likely source of

infection for the child.

Done; please see above.

482 cause rather than originate. You need to present data on relapse frequency for your

setting if you can.

Done (L 206-209).

484 – 487 This issue here is that the mother should be treated, and there are restrictions

around this. Your data suggests that children are ok up until one year.

Mothers with vivax malaria are routinely treated in Brazil. The former WHO recommendation that lactating women should not be given primaquine is no longer valid and has never been strictly adhered to in Brazil. The vast majority of study mothers did receive the usual chloroquine-primaquine treatment; no restrictions apply to mothers. However, infants <6 months of unknown G6PD status are not prescribed primaquine in Brazil. This restriction does apply to young children but appears to have no major public health consequences in our setting given the rarity of malaria in this age group.

Limitations: mention sample size

Ok, done L 786-789);

Missing data

Actually, there are very few missing data. The main limitation is the lack of follow-up of children living in remote rural areas, who tend to be more exposed to malaria (acknowledged in L 786-787).

Multiple comparisons

I missed the point here. We tested the following outcomes: (a) malaria incidence in children, (b) time to the first vivax malaria episode in children, (c) time to the first vivax malaria episode in mothers, and (d) anemia. Multivariable models were used to identify independent predictors of each outcome while adjusting for all other covariates. There is no parallel testing of several exposures in separate models with the same outcome, a situation that would require adjustment for multiple comparisons.

Single time point of screening for infection in all women (delivery)

Please see L 203-206: “The diagnosis of malaria in pregnancy was defined by combining antenatal diagnosis by microscopy of Giemsa-stained capillary blood thick smears prepared during sick visits to public clinics and species-specific real-time PCR [20] carried out on venous blood samples collected at delivery [21].” Mothers were not tested for malaria parasites only at delivery; indeed, we have the information about malaria cases diagnosed during sick visits throughout pregnancy.

Lack of documentation of radical cure in mothers or infants.

Case notification records does include the treatment used (L 259-263). All mothers with postpartum malaria were prescribed radical cure (i. e., chloroquine plus primaquine). All children older than 6 months were similarly prescribed radical cure. Because treatment was unsupervised, adherence may not be very high (acknowledged in L 75-760).

This issue with not having much P. falciparum is not a major limitation – your strength is that you are looking at Pv, for which there is not much such data.

Pf would be an interesting comparator in many analyses. For example, is Pf-associated anemia more or less common and severe than that associated with Pv?

**Conclusion**

The first two sentences should probably go into introduction rather than be named here. This

section focusses on the findings of this analysis.

Done.

508-512 – split sentence into 2

Done.

512 – you are not showing the data on anaemia and impaired infant growth so I would state

‘which has been associated with deleterious impacts on child growth and development’.

Offer a way forward how you suggest the problem can be prevented, based on your findings.

What can be done to protect young children to get P. vivax? Etc.

Done.

**Table 1**

Footnote a – discuss this in more detail in the methods section. There you mention creating a

variable for missing data, so this is confusing.

The number of children with missing information may be easily computed from data shown in Table 1. There are 1,494 children included in the analysis (as stated in Methods and Table 1 heading). We have wealth index information, for example, for 1,426 children (please see data in Table 1), indicating the this information was missing for 1,494 – 1,426 = 68 children (4.5% of the total). These 68 children were included in the missing-value category (not “variable for missing data”). The advantage this method has over other ways of dealing with missing values, such as imputation, is that it makes no assumption about the missing values (i.e., be it random or not random or replacing the missing value with an estimate). This is a standard procedure in multivariable statistical analysis.

This table/analysis could including maternal postpartum infection as a variable.

Please see above.

Figure 3 – here you used episodes per person-month – what do you use to calculate the IRRs.

Define incidence in your figure cap.

Done.
